# An Analysis of Visibility and Anatomic Variations of Mandibular Canal in Digital Panoramic Radiographs of Dentulous and Edentulous Patients in Northern Iran Populations

**Published:** 2016-06

**Authors:** Somayeh Nemati, Anahita Ashouri Moghadam, Zahra Dalili Kajan, Seyedeh Tahereh Mohtavipour, Hasan Amouzad

**Affiliations:** 1 Dept. of Dentomaxillofacial Radiology, Dental Faculty, Guilan University of Medical Sciences, Rasht, Iran.; 2 Dept. of Periodontology, Dental Faculty, Guilan University of Medical Sciences, Rasht, Iran.; 3 Dentist, Rasht, Iran.

**Keywords:** Panoramic Radiography, Digital Radiography, Edentulous, Jaw, Mandible

## Abstract

**Statement of the Problem:**

Insufficient information about the anatomical positions and structure of mandibular canal provokes unwanted damage to this important structure of mandible.

**Purpose:**

The aim of this study was to determine the visibility and anatomical variations of mandibular canal in digital panoramic radiographs of dentulous and edentulous patients in a sample of Iranian population.

**Materials and Method:**

In this retrospective-analytical research, 249 digital panoramic radiographs in dentulous group and 126 in edentulous group were studied by an expert oral and maxillofacial radiologist. In both groups, the visibility of canal borders in anterior, middle, and posterior areas were examined. In dentulous group, the distance between the canal and apex of the first and second molars were measured. Canal-to-alveolar crest distance and lower mandibular border was measured in three different points for both groups. Finally, the upper-lower positions of canals were determined.

**Results:**

In both groups, most visibility occurred in 1/3 of posterior and the least visibility was detected in 1/3 of anterior, with the intermediate being the most visible part (Type 2). There was no significant difference between the left and right sides in all cases. In dentulous group, no correlation was found between the visibility, age, and gender (*p*> 0.05); however, canal position was related to gender (*p*= 0.03 and *p*= 0.04 in right and left sides, respectively). High position was more frequent in females and intermediate position was more common in males. In edentulous group, no correlation was found between age, gender, and canal position (*p*> 0.05).

**Conclusion:**

The most visibility of mandibular canal was in its third posterior and the least was in its third anterior part. Although the middle position of canal was more frequently visible than the high position in this study, it does not refute the possibility of damaging the mandibular canal in critical surgeries.

## Introduction

Radiographic diagnosis of a disease requires a precise knowledge of anatomic landmarks and natural structures. This kind of diagnosis cannot happen without considering the variations and alterations of natural anatomical structures.[1]The radiographic images and width of the mandibular canal show some variations among the patients. Sometimes the borders are only seen partially or not at all.[2] In maxillofacial surgeries, mandibular canal is considered a reference anatomic structure. Extracting the mandibular third molar, implant surgeries, orthognathic surgeries, and fixing the jaw fractures are cases with the high risk of damage to the mandibular canal and inferior alveolar nerve.[3] Proximity of the first and second molar root to the mandibular canal can cause injury to inferior alveolar nerve while extracting these teeth.[4] Inferior alveolar nerve may get traumatized in its intraosseous pathway. The most common place of injury is the third molar of lower jaw. Extracting the impacted third molar may result in nerve crushing or damage.[5] One side effect is dysesthesia, which includes paresthesia and anesthesia. This damage is related to deep impaction of tooth and proximity of roots to inferior alveolar nerve.[4, 6] Inferior alveolar nerve can get damaged during endodontic or even orthodontic treatments. Over instrumentation or overfilling in mandibular premolar or molar teeth during endodontic treatment can cause nerve damage. Orthodontic movements of posterior mandibular teeth can impose pressure on inferior alveolar canal and even paresthesia.[7]

Histologic studies have shown that the path of inferior alveolar nerve is typically in form of a main trunk (with sub-branches) toward the teeth apex in mandible. But there are some smaller parts of inferior alveolar nerve that are almost parallel to the main branch. In some cases, they are so eminent that are considered as second mandibular canal. Two-branch mandibular canal (bifid) can be seen on panoramic radiographs and cone beam computed tomography (CBCT) images. Patients with bifid canals are at risk of inadequate anesthesia or some problems with jaw surgeries including dental implants.[2] Langlais *et al.*[8] divided the two-branch mandibular canal into four groups, regarding their anatomical position. Accordingly, in type 1, two-branch canal stretches toward the third molar or its surrounding either one-sided or two-sided. In type 2, the two-branches of canal are rejoined in ramus either one-sided or two-sided. Type 3 is the combination of type 1 and 2. In type 4, two canals originate from separate mandibular foramen and join to form a large canal. Nortje *et al.*[9] divided the mandibular canal in three groups based on its superior-inferior position. In superior position, a single canal is in contact with the apex of the first and second permanent molars or at its 2-mm distance. In case of their loss, the canal position should be considered in contact with the approximate position of the root apex of the first and second molars (based on the neighboring tooth). Inferior position includes a single canal in contact with or at a 2-mm distance from the inferior border of mandibular cortical plate. Middle position is between the superior and inferior positions. Considering the great usage of panoramic radiography in dentistry, interpatient variability of mandibular canal, and lack of any previous similar study, the present study was designed to assess the visibility and anatomical variations of mandibular canal in dentulous and edentulous patients and to compare these variations with respect to the side, age, and gender in Guilan Dentistry Faculty, Rasht, Iran.

## Materials and Method

In this retrospective descriptive-analytical study, 375 panoramic radiographs which were comprised of 249 dentulous (124 male and 125 female) and 126 edentulous (80 males and 46 females) cases were analyzed; the radiographs were taken from a sample population in Northern Iran. These radiographs were collected from the Department of Oral and Maxillofacial Radiology at Dentistry Faculty of Guilan University of Medical Sciences, Rasht, Iran (Northern Iran), from November 2012 to July 2014. They were prescribed by colleagues in other departments for different purposes. Before conducting this investigation, we originally obtained the approval of Institutional Ethics Committee of Guilan University of Medical Sciences Research Foundation (#IR.GUMS.REC.1394.446) to ensure the compliance of our protocol with the guidelines of the Declaration of *Helsinki *and Tokyo for humans. We also obtained written consent from the participating patients to use their panoramic images for this study. All radiographs were taken by using a digital panoramic imaging system (Soredex Cranex-D^TM^; Finland). They were analyzed on a 14" HD LED (1366×768) by using Scanora software version 4.3.1 with similar magnification. All panoramic radiographs were displayed in same scale (true size) without any manipulation for digital zooming in software.

The mandibular canal was evaluated in all radiographic images with the same digital processing with no alteration to the primary processing. Calibration was done in all measurements. This study was performed for all cases on both left and right sides. Radiographs were divided into two groups in which group 1 were dentulous and group 2 were edentulous cases, respectively. In group 1(dentulous group), the inclusion criteria were being an adult of over 18 years old and having the second premolar and the first and second molar on both sides. The exclusion criteria were having periodontal disease and bone degeneration in mandible, pathologic radiolucent or radiopaque lesion in mandible, radiolucent lesion in peri-apex of the first and second mandibular molars, fractures involving the mandibular canal, alveolar bone loss in the first and second molars, as well as technical errors and improper quality of radiographs. 

**Figure 1:**
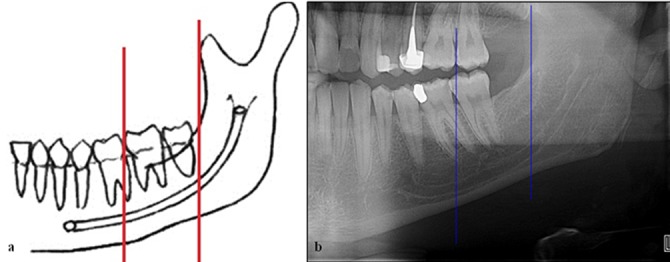
a: Schematic view of a dentulous patient dividing the mandibular canal by vertical lines to 3 sections (posterior, middle and anterior). b: Panoramic view of a dentulous patient.

Canal visibility was among the investigated features in this research. Visibility of the upper and lower mandibular canal was investigated in 3 areas of anterior, middle, and posterior. By using the software, a vertical line was drawn in the first molar and another one along the ramus anterior border. So, the mandibular canal was divided into posterior, middle, and anterior areas (Figures 1a and b).

**Figure 2:**
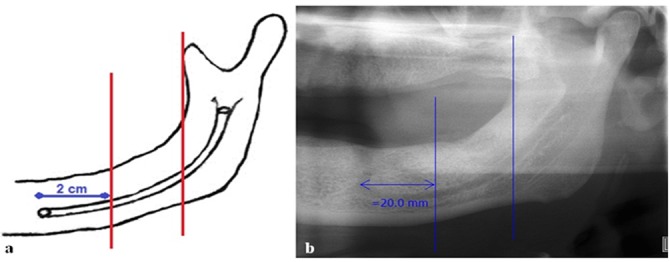
a: Schematic view of edentulous patient; the vertical lines divide the mandibular canal into 3 sections (posterior, middle and anterior). b: Panoramic view of an edentulous patient.

Visibility was considered positive if the superior and inferior borders of mandibular canal were visible in at least 75% of the canal path. (Figure 2)

If the superior and inferior borders of the canal were clearly visible in the anterior and middle areas of the radiograph, the 10 following distances were measured on each side. Based on Nortje *et al.*’s[9] classification, superior-inferior position of the canal was marked as high (type 1), intermediate (type 2), or low (type 3) position:

1. From the superior border of canal to alveolar crest between the first molar and second premolar (point A)
2. From the superior border of canal to alveolar crest between the first and second molar (point B)
3. From the superior border of canal to alveolar crest in the second molar distal (point C)
4. From the inferior border of canal to inferior border of mandible between the first molar and second premolar (point A´)
5. From the inferior border of canal to inferior border of mandible between the first and second molar (point B´)
6. From the inferior border of canal to inferior border of mandible in the second molar distal (point C´)
7. From the superior border of canal to apex of mesial root in the first molar (point D)
8. From the superior border of canal to apex of distal root in the first molar (point E)
9. From the superior border of canal to apex of mesial root in the second molar (point F)
10. From the superior border of canal to apex of distal root in the second molar (point G)

In group 2 (edentulous group), the inclusion criteria were complete loss of teeth in lower jaw and precise diagnosis of mental foramen on both sides of the mandible. Exclusion criteria were having radiolucent or radiopaque pathologic lesion in mandible, fractures involving canal, technical errors, diagnosis of improper quality in radiography, and visibility of the socket in newly-extracted tooth in alveolar crest of mandible.

**Figure 3:**
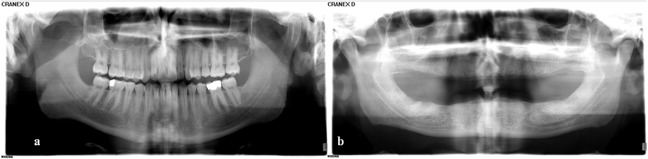
a: Visibility of all borders of the mandibular canal on both sides in a dentulous 24-year- old man. b: Visibility of all borders of the mandibular canal on both sides in a 65-year-old edentulous man.

Similar to group 1, visibility of the inferior and superior borders of mandible canal was studied. The point of differ to divide the canal into posterior, middle, and anterior regions was to draw a vertical line 2cm behind the center of mental foramen and another one along the anterior border of ramus (Figure 3).

In dividing the canal positions, inferior and intermediate positions were considered similar to classification of Nortje *et al.*,[9] but for the superior (type 1), a 10-mm distance to alveolar crest was considered type 1 canal because the minimum length of implant regardless of its diameter for replacing the posterior mandibular teeth is 10 mm.[10] This modification was done to deliberate the high risk of implant and surgery procedures for these people. In radiographs which both inferior and superior borders of the canal were thoroughly visible, the following 6 distances were measured:

1. From the superior border of canal to alveolar crest 1 cm behind the mental foramen (point A)
2. From the superior border of canal to alveolar crest 2 cm behind the mental foramen (point B)
3. From the superior border of canal to alveolar crest 3 cm behind the mental foramen (point C)
4. From the inferior border of canal to inferior border of mandible 1 cm behind the mental foramen (point A´)
5. From the inferior border of canal to inferior border of mandible 2 cm behind the mental foramen (point B´)
6. From the inferior border of canal to inferior border of mandible 3 cm behind the mental foramen (point C´)

After collection of data, all statistical analyses were performed by SPSS software (version 21), using chi-square and independent t test. The statistical significance was set at *p*<0.05 for all tests.

## Results

In dentulous group, the mean age was 26.37±5.87 with the minimum being 18 and maximum 52. On both left and right sides and in both genders, the most canal visibility was in one-third of posterior and the least was in one-third of anterior. In all the three parts of the canal, inferior border was more visible than the superior (Table 1). The most common visible position of the canal was intermediate (Type 2) followed by high position (Type 1), with no canal being in low position (Table 2).

**Table 1:** Frequency distribution of canal visibility in different portions of mandibular canal on the left and right sides (dentulous group)

**Mandibular canal visibility**	**Right side**		**Left side**		**P value in chi-square**
	**Number**	**(%)**	**Number**	**(%)**	
Superior border in anterior	107	43	100	40.2	0.00
Superior border in middle	124	49.8	128	51.4	0.00
Superior border in posterior	248	99.6	247	99.2	0.00
Inferior border in anterior	191	76.7	197	79.1	0.00
Inferior border in middle	227	91.2	223	89.6	0.00
Inferior border in posterior	248	99.6	249	100	0.00

**Table 2:** Frequency distribution of mandibular canal position on the right and left sides in men and women (dentulous group)

**Mandibular canal position**	**Men**		**Women**		**Total**		**P value in chi-square**
	**Number**	**Percentage**	**Number**	**Percentage**	**Number**	**Percentage**	
High position, right	2	6.1	7	25.9	9	15.0	0.03
Intermediate position, right	31	93.9	20	74.1	51	85.0	
High position, left	1	3.0	5	18.5	6	10.0	0.04
Intermediate position, left	32	97.0	22	81.5	54	90.0	

The most distant point to crest and inferior border of the mandible were point A and point C´, respectively.

The longest distance between the canal and apex of the first and second molars belonged to mesial root of the first molar and shortest distance was seen in distal root of the second molar (Table 3).

**Table 3:** Measurement of mandibular canal distance to inferior border of mandible, alveolar crest and root apices in 10 points (dentulous group)

**Measured** Distances^*^	**Right** **Mean** (±SD)^**^	**Left Mean** **(±SD)**	**P value in independent** **t-test**
1 (point A)	2.70±21.38	2.71±21.30	0.66
2 (point B)	2.70±19.31	2.53±19.61	0.08
3 (point C)	2.25±15.14	2.42±15.57	0.06
4 (point A´)	1.73±6.42	1.80±6.50	0.60
5 (point B´)	1.48±5.42	1.52±5.34	0.51
6 (point C´)	2.02±7.39	1.89±7.25	0.50
7 (point D)	2.62±6.57	2.60±6.47	0.64
8 (point E)	2.46±5.97	2.52±6.26	0.23
9 (point F)	2.57±3.97	2.73±4.39	0.05
10 (point G)	2.28±3.44	2.41±3.69	0.09

*: For more information, see the text.

**: Standard deviation

No significant difference was found in canal position and the measured distances on the left and right sides (Table 1, Table 3). There was significant correlation between canal visibility on the left and right sides, based on chi-square test (*p*= 0.0001). According to the results of in dependent t test, no correlation was found between the canal visibility and the patients’ age on both sides (*p*> 0.05). Independent t test showed that the canal position was not related to age (*p*= 0.3, 0.95 on the right and left sides, respectively). Yet, canal position was related to gender so that on both sides, high position was more frequent in women than men and intermediate position was more common in men (Table 2, chi-square test). Independent t test also revealed that the measured distances in this group were not related to age (*p*> 0.05). But on both sides, the canal distance to alveolar crest in points B and C, canal distance to mandibular border in A´, and canal distance to apex of the first and second molar were significantly longer in men (*p*< 0.05, independent t-test).

In edentulous group, the mean age was 58.80± 10.46 with the youngest being 30 and the oldest 90. Similar to the previous group, for both genders and on both sides of the jaw, the most visibility of canal was in one-third of posterior and least was in one-third of anterior. In all the three parts of the canal, inferior border was more visible than the superior (Table 4).

**Table 4:** Frequency distribution of canal visibility in different portions of mandibular canal on the left and right sides (edentulous group)

**Mandible canal**	**Right side**		**Left side**		**P value in chi-square**
	**Number**	**Percentage**	**Number**	**Percentage**	
Superior border in anterior	40	31.5	45	35.5	0.00
Superior border in middle	43	3.33	49	38.1	0.00
Superior border in posterior	119	94.4	116	92.1	0.00
Inferior border in anterior	86	63.8	82	65.1	0.00
Inferior border in middle	98	77.8	98	77.8	0.00
Inferior border in posterior	123	97.6	120	95.2	0.00

The most common position of the canal was intermediate (Type 2) and then high position (Type 1), with no canal being in low position (Table 5).

**Table 5:** Frequency distribution of mandibular canal position on the right and left sides in men and women (edentulous group)

**Canal Positions**	**Men**		**Women**		**Total**		**P value in chi-square**
	**Number**	**Percentage**	**Number**	**Percentage**	**Number**	**Percentage**	
High position , right	6	3.33	2	40.0	8	39.1	0.28
intermediate position, right	6	3.33	2	40.0	8	39.1	
High position, left	5	27.8	1	20.0	6	26.1	0.72
intermediate position, left	13	72.2	4	80.0	17	73.9	

Based on the results of chi-square test, there was no significant difference between the canal visibility and canal position on left and right sides with a significant correlation on both sides (*p*= 0.0001). Moreover, there was no significant difference between the measured distances on the left and right sides (*p*> 0.05) based on independent t test (Table 6).

**Table 6:** Measurement of mandibular canal distance to inferior border of mandible and alveolar crest in 6 points (edentulous group)

**Measured distances** ^***^	Right Mean(±SD)****	**Left Mean(±SD)**	**P value in independent t-test**
1 (point A)	4.54±12.19	4.44±12.41	0.66
2 (point B)	4.06±10.70	3.87±10.83	0.79
3 (point C)	3.15±9.60	2.94±9.88	0.55
4 (point A´)	1.43±7.93	1.36±8.26	0.14
5 (point B´)	1.40±7.09	1.56±7.67	0.13
6 (point C)´	1.72±7.29	1.92±7.84	0.57

*** For more information, see the text.     **** Standard Deviation.

However, chi-square test showed a correlation between canal visibility and gender on both sides, that is, the superior border visibility in all the three parts of canal and inferior border visibility in one-third of anterior and one-third of middle was more in men than women (*p*< 0.05). By using independent t test and chi-square analysis, no significant correlation was found between age and gender with the canal position and measured distances on both sides (*p*> 0.05).

## Discussion

In recent years, different studies have focused on normal landmarks and their natural structures for better identification of pathological lesions and diagnosis, and subsequently a better treatment plan. In this regard, neurovascular bundle in lower jaw is considered to be one of the most important and concerned landmarks.[11-12]

It is necessary to have adequate and proper information about the variations in mandibular canal path and its topography.[13] Panoramic view is one of the most common radiographies in dentistry used by many dentists as a routine diagnostic imaging method in jaw problems and traumas.[14] In the present study, most visibility of inferior and superior borders in both groups, for both genders and on both sides, belonged to one-third of the posterior and the least visibility was in the one-third of anterior. These results may be due to changes in the pathway of mandibular canal to the buccal side before opening in mental foramen[15] which causes the anterior part being less clearly visible. This is in accordance with Angelopoulos *et al.*’s study[15] which compared the mandibular canal visibilities in CBCT with panoramic radiographs made by PSP (DENOPTIX) and CCD (DIMAX) digital detectors. The canal was most visible in CBCT followed by panoramic CCD (DIMAX), and least visible in images made by PSP (DENOPTIX). In both CBCT and panoramic radiographs, the most visibility belonged to posterior one-third of the canal and the least was in its anterior part. Moreover, in the current study, in both groups and both genders, the visibility of inferior border was higher than the superior border; this is similar to Pria *et al.*’s[16] findings.

In the present study, in dentulous group, 15% of the canals on the right side and 10% of the canals on the left side were in high position (type 1). In dentulous group, in high position of the canal and in both sides, women had a relative superiority to men. This matter should always be remembered when observing the panoramic radiographs of women due to the high possibility of damaging to the canal while performing surgical, endodontic, orthodontic, and dental implant procedures. In groups, genders, and sides, the most common position of the canal in superior-inferior dimension was the intermediate position (type 2) and the high position took the second rank. 

In dentulous group, on both sides, the intermediate position of the canal was the most common position in men. Contrary to our results, Nortje *et al.*’s[9] study reported the least visibility in intermediate position (3.3% of the canals). This can be due to racial and sample-size differences. In the current study, in both groups, genders and sides, no canal was visible in low position (type 3); while, Nortje *et al.*[9] reported 48.9% of canals to be in low position. The difference can be attributed to racial and sample-size differences as well as the quality of panoramic images in detecting the mandibular canal visibility.

This study showed that in dentulous group, for both men and women and on both sides, the distance between the superior border of canal and alveolar crest was the longest between the second premolar and first molar. This is comparable to a research reported by Liu *et al.*[17] In their study, the longest distance between the canal and alveolar crest was in second premolar area, but, quantitatively, it was less than what current study obtained (17.76±2.24mm).

The present study found the shortest distance between inferior border of canal and inferior border of mandible to be between the first and second molars. Again, this is comparable to Liue *et al.*’s findings.[17] In their study, the least distance between the canal and inferior border of mandible was in the first molar area, but quantitatively, it was a little more that the measure of our study (7.56±1.62mm).

 In dentulous group of our study, the distance between the inferior border of canal and inferior border of mandible in the second molar distal was the longest. In both groups, no significant difference was detected between the distances of inferior border of canal to inferior border of mandible on both sides of mandible. However, there was a significant difference between males and females; the distance of canal to inferior border of mandible between the first molar and second premolar was longer in men. 

**Figure 4:**
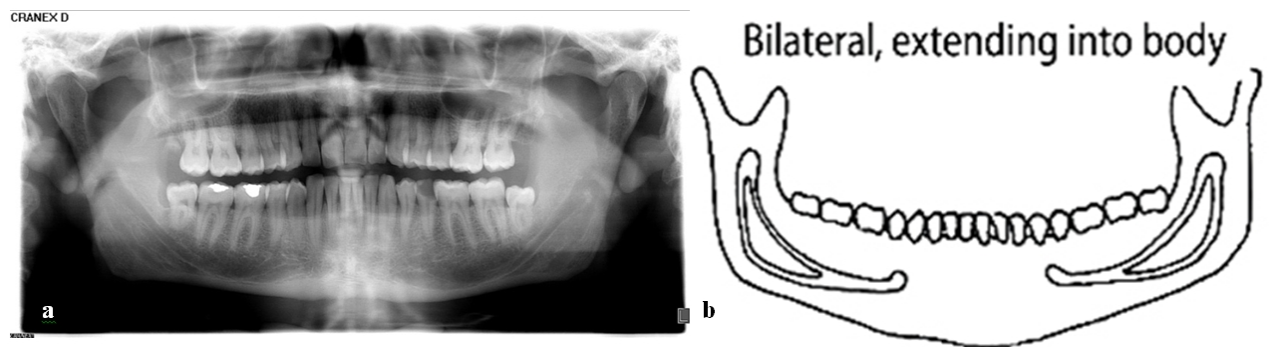
a: Bilateral bifid mandibular canal in a 33 year- old male. b: a type II of two-branch canals based on Langlais *et al.*’s categorization

The mean distance from the superior border of canal to the apex of mesial root of the first molar was measured to be 6.57±2.62mm, and to the apex of distal root of the first molar was 5.97±2.46mm. These findings were similar to Ghanim’s[18] research on bisecting periapical radiographs. Their study reported the above-mentioned distances to be 6.16±2.84mm and 5.63±2.74mm. It must be taken into account that in surgical, periodontal, endodontic, and other treatments, the chance of damaging the second molar distal root is higher than the first molar mesial root.[19] It is important to note that inadequate anesthesia may be possible with any bifid mandibular canal, especially when there are two mandibular foramens.[20] In dentulous group of current study, only one case of bifid mandibular canal was observed on panoramic radiography, which showed 0.4% prevalence. This is close to Sanchis *et al.*’s[21] findings that reported this prevalence to be 0.35%. The two-branch canal in the present study was a two-way type and belonged to a 33 year-old male. This canal started on both sides from the inner surface of ramus and then divided in two separate branches, then, these branches joined in molar area and formed a single canal and ended in mental foramen. Based on the classification of Langlais *et al.*[8] it was a type II of two-branch canals (Figures 4a and b).

Kumar *et al.*[22] evaluated the morphological variations of the mandibular canal in panoramic radiographs. In their study, the most common variations were bifid mandibular canal (4.3%) and double mandibular canal (4%) respectively. In comparison with the present study, the prevalence of bifid mandibular canal was higher. The authors elucidated that the disparity and overestimation of their results from the previous studies could be due to differences in the study design and image interpretation criteria.

Due to the limited number of panoramic images of edentulous patients in the new campus of Dentistry Faculty of Rasht, fewer cases were put in edentulous group than in dentulous group. Moreover, because this study was retrospective, it was not possible to precisely evaluate the time of teeth extraction and its effect on the distance of mandibular canal to alveolar crest. This study was the only one investigating the visibility and variations of mandibular canal anatomy in edentulous versus dentulous patients in a small sample population of Northern Iran and because of individual differences it could not be extended to the rest of the population. Future studies, using CBCT images with a larger sample size in both groups are suggested to achieve more precise results. Finally, since the panoramic images in this survey were used without changing the digital processing conditions, a research on the outcome of digital processing on mandibular canal visibility is necessary. The findings of such studies may impact our findings. 

## Conclusion

In both dentulous and edentulous groups, the most visibility occurred in 1/3 of posterior, the least was in 1/3 of anterior, and intermediate position was the most common (Type 2). Although the middle position of canal was more frequently visible than the high position in this study, it does not refute the possibility of damaging the mandibular canal. Therefore, it is suggested that dentists analyze panoramic radiographs more carefully and use CBCT in high-risk patients. Dentists must carefully analyze panoramic images in high-risk surgeries and dental implant planning. 
